# Community abstracts: coming soon!

**DOI:** 10.1093/jamiaopen/ooz070

**Published:** 2020-01-20

**Authors:** Indra Neil Sarkar

**Affiliations:** Center for Biomedical Informatics, Brown University, Providence, Rhode Island, USA

When *JAMIA Open* was launched, we aspired to be a leading peer-reviewed publication that supported: (1) data openness and (2) community impact. The goal of data openness was rapidly achieved through partnership with Dryad (https://datadryad.org/journal/2574-2531). The second goal of community impact was envisioned as community-facing abstracts that would accompany each paper. I am pleased to report that starting in 2020, all accepted manuscripts will have community abstracts published alongside the traditional publication. It is hoped that this new feature will encourage patients and community members to better understand the work that is published in *JAMIA Open*. It is also expected that this will force us all to reflect on the real-world impact of our work. 

Mechanically, we will require community abstracts only after a manuscript has been accepted for publication. Acknowledging that this may be new for many authors, we will provide guidance on how to develop community abstracts. I will also be inviting authors of all papers that have been published to date to also supply community abstracts, which will be made available in a single publication that links back to the original papers.

What’s next? After we have implemented the community abstract, we will then focus attention to provide mechanisms for supporting live content (eg, code associated with a paper). It is expected that this feature will further enhance the reader experience and also promote reproducibility.

Going into the next year, *JAMIA* and *JAMIA Open* will have a cross-journal special focus issue on the Unified Medical Language System (UMLS). *JAMIA Open* will consider Application Note, Database Note, and Patient Perspective articles. This partnership reflects the continued synergy between the journals and the collaborative spirit of our community. More information about the special focus issue can be found here: https://academic.oup.com/jamia/pages/unified_medical_language_cfp.

We are still in the process of selecting the next cohort of Editorial Board Members, who will join the *JAMIA Open* in 2020. Editorial Board service is essential to the success of any journal, and we have benefitted from the dedicated inaugural cohort. I am also grateful to the community of *ad hoc* reviewers who provide the essential evaluation of submissions and help guide the Associate Editors and myself to identify the very best work to publish.

As you read this issue of *JAMIA Open*, take a moment to consider how the presented work advances health care. And, as always, I also ask that you share your favorite papers on social media.

